# 伴有t(16;17)染色体异常的APL样白血病1例报告并文献复习

**DOI:** 10.3760/cma.j.cn121090-20240118-00033

**Published:** 2024-09

**Authors:** 倩 王, 天鑫 吕, 昊 艾, 晓东 吕, 青松 尹

**Affiliations:** 郑州大学附属肿瘤医院（河南省肿瘤医院），郑州 450008 The Affiliated Cancer Hospital of Zhengzhou University & Henan Cancer Hospital, Zhengzhou 450008, China

## Abstract

变异型急性早幼粒细胞白血病（APL）及APL样白血病均是APL中的少见类型，其中t（16;17）染色体异常更为罕见。本文报道1例伴有t（16;17）染色体异常及骨骼、淋巴结及中枢神经系统侵犯的APL样白血病患者，经过多周期化疗后获得完全缓解，随后进行造血干细胞移植，并进行文献复习。

## Abstract (en)

Variant acute promyelocytic leukemia（APL）and APL-like leukemia are rare types of APL, with t（16;17）chromosome abnormality being even rarer. An APL-like patient with t（16;17）chromosome abnormality, which was characterized by bone, lymph node, and central nervous system involvement, was admitted to our hospital. He achieved complete remission after several cycles of chemotherapy and subsequently underwent hematopoietic stem cell transplantation. Furthermore, the diagnosis and treatment of this patient were reported and a literature review was conducted.

急性早幼粒细胞白血病（APL）是急性髓系白血病（AML）中预后相对较好的一种类型，长期生存率可以达到90％以上，其中，变异型APL具备APL的临床和细胞形态学表现，但细胞遗传学或分子生物学未表现经典APL的15及17号染色体易位，多为11号、5号、1号等染色体的特定位点与17号染色体长臂的1区2带或2区1带发生易位。我们收治1例16及17号染色体易位的APL样白血病患者，现将诊疗经过报道如下，并进行文献复习。

## 病例资料

患者，男，8岁，2021年10月无诱因出现左侧腘窝处疼痛并间断发热，无皮肤黏膜出血表现，无头晕、乏力、黑矇等贫血表现，于某骨科医院就诊，2021年12月9日左下肢平扫+增强磁共振（MRI）提示左股骨远端干骺端及胫腓骨近端干骺端异常信号影，考虑恶性占位性病变。12月10日血常规示：WBC 6.49×10^9^/L，HGB 106 g/L，PLT 402×10^9^/L，D-二聚体10 mg/L，纤维蛋白原降解产物（FDP）115.69 µg/ml，纤维蛋白原正常。左下肢骨髓活检病理考虑“粒细胞肉瘤”，为进一步诊治于2022年1月4日入我院，入院时有左侧腘窝处疼痛，无发热及其他症状。1月4日进行骨髓穿刺送检骨髓细胞形态学、流式免疫表型、二代测序筛查融合基因及突变基因、染色体。骨髓细胞形态回报：有核细胞增生极度活跃，可见大量异常早幼粒细胞，占89.2％，其他阶段粒细胞少见（图1）。外周血涂片：异常早幼粒细胞占16％。1月5日血常规：WBC 4.8×10^9^/L，HGB 114 g/L，PLT 175×10^9^/L，D-二聚体27.08 mg/L，纤维蛋白原1.38 g/L。患者体重29 kg，体表面积1.11 m^2^，因患者骨髓形态符合APL，1月5日开始口服全反式维甲酸（ATRA）10 mg/次，每日2次。骨髓检查陆续回报：1月6日回报骨髓细胞实时定量PCR检测PML-RARα融合基因阴性，白血病免疫分型：异常髓系原始细胞占有核细胞的55.09％，SSC值较高，表达CD117、CD38、CD33、CD13、cMPO，弱表达CD7、CD123，不表达CD34、HLA-DR。外院髂骨及骨髓活检标本病理会诊示：（左髂骨）AML/髓系肉瘤累及骨髓。免疫组化：CD3（−），CD4（−），CD8（−），CD33（+），CD34（−），CD117（弱+），CD68（+），CD123（−），NSE（−），CD56（−），CD30（−），LCA（−），CK（−），CD99（+），MPO（+），EBER（−）。FISH检测回报PML-RARα融合基因阴性。1月7日行骨盆及左膝关节平扫+增强MRI提示：左侧髂骨、骶骨左侧份、左侧髋臼骨及左侧耻骨骨质异常信号改变伴周围肿块，考虑恶性，邻近腰大肌、臀肌信号不均，水肿可能，局部受侵待排；双侧髂血管走形区、左侧腹股沟区及所示腹膜后多发肿大淋巴结，大者短径约19 mm；右侧髂骨斑片状强化影，双侧股骨下段、胫腓骨上段异常信号（图2）。1月7日开始应用亚砷酸（ATO）0.16 mg·kg^−1^·d^−1^治疗，1月6至9日，WBC波动于（4.8～8.7）×10^9^/L，PLT由175×10^9^/L逐渐下降至125×10^9^/L，FDP由最高203.9 µg/ml下降至23.1 µg/ml，D-二聚体由最高30.64 mg/L下降至3.63 mg/L。由于未检出PML-RARα融合基因，且血常规未呈现因早幼粒细胞分化导致的白细胞升高，考虑患者并非经典型APL，1月9日停用ATO及ATRA，开始IA方案化疗，具体用药：伊达比星10 mg/d第1～3天，阿糖胞苷100 mg/d第1～7天。1月11日染色体结果回报（图3）：染色体核型为46,XY,t（16;17）（q11;p12）[7]/92,XXYY,idem×2[3]。1月12日68项AML相关基因突变二代测序结果回报：FLT3-TKD（转录本ID NM-004119.3，核苷酸改变c.2510_2512delTGA，氨基酸改变p.Met837del）突变频率36.71％。1月17日探针捕获法二代测序查血液病融合基因高通量筛查回报：未检出相关融合基因。进一步使用患者骨髓样本进行全基因组深度测序（包括全基因组范围内的大片段拷贝数变异、单核苷酸变异、结构变异），检出多个融合基因，但未检测到与APL相关的融合基因，另可见16q−，17p−，17q+的拷贝数变异。最终诊断为APL样白血病伴多发骨、淋巴结累及。2022年1月18日（化疗结束后第3天）骨髓细胞形态学检测：增生减低，异常早幼粒细胞为71.5％。外周血涂片可见21％异常早幼粒细胞。1月25日（化疗结束后第10天）复查骨髓细胞形态学：增生明显减低，异常早幼粒细胞为28.0％。外周血涂片未见异常早幼粒细胞。1月25日给予阿扎胞苷0.1 g，皮下注射，第1天；索拉非尼0.2 g/次，口服，每日2次；维奈克拉100 mg/d×14 d。2022年1月30日（化疗后第15天）胸骨骨髓细胞形态学检测：增生明显减低，异常早幼粒细胞19.5％。2月14日（化疗后第30天）髂后骨髓细胞形态学检测：增生明显减低，偶见异常早幼粒细胞。流式细胞术微小残留病检测：共获取有核细胞约5×10^5^个，可疑髓系原始细胞占0.19％，表型为CD34^+^CD117^+^CD13^dim+^CD33^+^CD7^−^HLA-DR^+^CD38^+^CD15^−^CD64^−^CD19^−^CD56^−^CD45^dim+^。外周血涂片未见异常早幼粒细胞。染色体为正常核型。2月16日进行腰椎穿刺+三联鞘内注射化疗，脑脊液形态可见异常早幼粒细胞（图4），脑脊液流式免疫表型未见异常。2月16日开始口服维奈克拉100 mg/d×14 d+皮下注射阿扎胞苷80 mg/d×7 d，2月21日、23日腰椎穿刺+三联鞘内注射化疗，脑脊液见异常早幼粒细胞，免疫表型未见异常。2月28日及以后多次复查脑脊液形态未见异常白细胞，腰椎穿刺时均给予三联鞘内注射化疗。2022年3月15日、4月18日、5月19日、6月30日复查骨髓细胞形态学均未见异常早幼粒细胞，流式细胞术微小残留病检测均为阴性，FLT3-TKD一代测序为野生型。3月22日、4月21日分别给予中剂量阿糖胞苷（2 g/次每12 h 1次，第1、3、5天）化疗。5月23日给予多柔比星脂质体注射液40 mg第1天；阿糖胞苷2 g/次，每12 h 1次，第1、3、5天；维奈克拉片100 mg/d×10 d化疗。分别于3月23日、5月12日、6月30日复查骨盆及膝关节增强MRI提示左侧髂骨、骶骨、左侧髋臼骨及左侧耻骨骨质异常信号影及双侧股骨下段、胫腓骨上段异常信号范围逐渐缩小。2022年7月4日进行骨盆至膝关节部位PET-CT检查，提示骶骨左半部分、左侧耻骨及左侧坐骨局部、左侧髂骨、双侧股骨下段、胫腓骨上段局部密度不均匀增高，部分代谢稍活跃［最大标准摄取值（SUVmax）1.3，肝脏SUVmax=1.34］（图5）。7月6日再次行脑脊液检查未见异常白细胞。考虑患者骨髓完全缓解状态，髓外病灶已消失，2022年7月14日给予全身照射+塞替哌+环磷酰胺方案预处理，2022年7月20日输注胞兄HLA 8/10相合外周血干细胞，每月骨髓检查提示完全缓解，流式细胞术MRD阴性，嵌合体为完全供者嵌合。2023年2月15日患者出现左侧臀部疼痛，可触及直径5 cm包块，2月17日超声引导下行左侧髋部皮下软组织穿刺，病理回报：镜下见多量退变坏死的小圆形肿瘤细胞残影巢状分布，考虑恶性肿瘤，MPO阳性。复查骨髓MRD仍为阴性，完全供者嵌合，考虑髓外复发。患者家属因经济原因要求出院，返回当地医院治疗后失访。

**图1:**
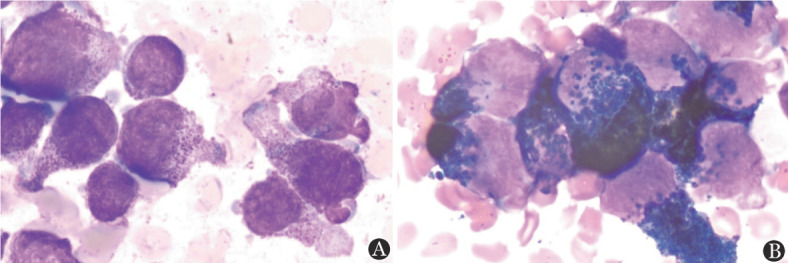
患者初诊骨髓细胞形态 **A** HE染色：异常早幼粒细胞明显增多，形态不规则，胞质浅蓝色，可见伪足状突起和内外胞质，胞质可见大量细小嗜天青颗粒，核形不规则，可见凹陷，扭曲或分瓣，染色质细致，核仁1个或多个；**B** POX染色强阳性

**图2:**
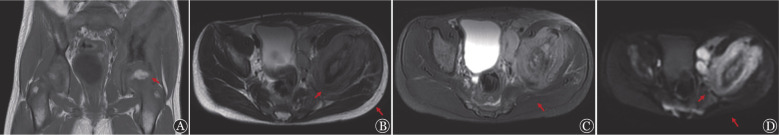
患者初诊髂骨MRI（箭头所指为左侧髂骨及髋臼骨骨质破坏，周围可见不规则肿块） **A** T1WI序列：低信号；**B** T2WI序列：低信号；**C** T2WI压脂序列：中等高信号影；**D** DWI序列：高信号影

**图3:**
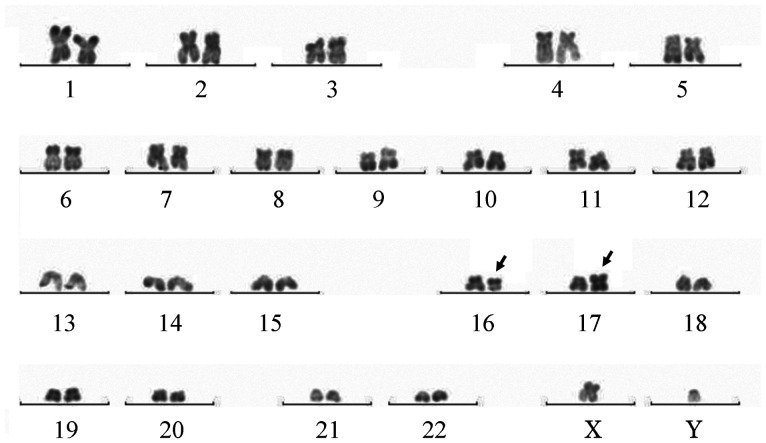
患者初诊骨髓染色体核型（箭头所指为16及17号染色体易位）

**图4:**
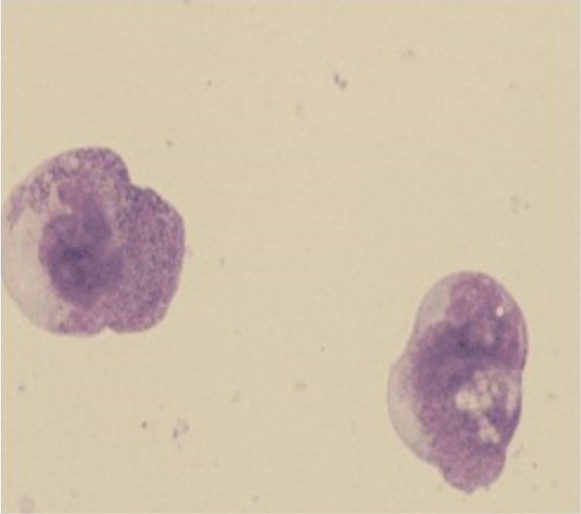
患者化疗后第32天（2022年2月16日）脑脊液甩片HE染色可见异常早幼粒细胞

**图5:**
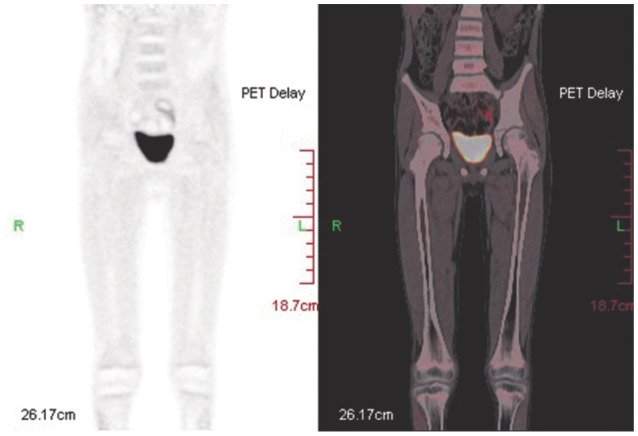
患者2022年7月4日局部PET-CT：骨盆左半部分及双侧股骨下段及胫腓骨上段部分代谢稍活跃

## 讨论及文献复习

变异型APL是APL中的一种少见类型，占所有APL的2％[1]。经典型APL由特征性的t（15;17）（q22;q21）染色体易位形成PML-RARα融合蛋白，抑制PML和RARα基因的正常功能，导致与造血分化相关的靶基因转录受到抑制，引起粒细胞分化受阻，促进白血病细胞增生，被认为是导致APL发病的关键分子[2]–[3]。ATRA和ATO可与PML-RARα融合蛋白结合，使其失效、降解，使得早幼粒细胞重新正常分化，并抑制其过度增殖。与经典型APL不同，变异型APL是其他的伴侣基因与RARα相互融合[4]，最常见的染色体异常为t（11;17）（q23;q21），其次为t（5;17）（q35;q21）、t（11;17）（q13;q21）、der（17）等[1],[4]，分别产生ZBTB16/RARα（即PLZF-RARα）、NPM-RARα、NuMA-RARα、STAT5b-RARα融合基因。变异型APL患者的临床表现、骨髓细胞形态学、免疫学特征与PML-RARα所致APL高度相似，但发病机制不同，对ATRA和ATO的治疗反应也不尽相同。PLZF-RARα与STAT5b-RARα、STAT3-RARα等类型的APL均对ATO和ATRA治疗反应不佳，大多数患者在短时间内复发，预后不良，其他变异型如NPM-RARα、NuMA-RARα、PRKAR1A-RARα等对ATRA的反应与经典APL类似，预后良好[5]。

APL样白血病的白血病细胞形态及免疫表型符合APL的特征，但缺乏涉及任何RAR的重排[6]–[7]，目前对于此类白血病多为个例报道[8]–[9]，尚不清楚这类病例是否具有共同的分子病理学及临床特征。本例患者以骨痛为首发表现，无明显出血倾向，初诊时血常规无明显异常，细胞形态可见胞质内大量细小嗜天青颗粒，“内外胞质”现象，POX染色强阳性，流式细胞术免疫表型SSC值高，MPO阳性，不表达CD34、HLA-DR，符合APL的细胞学及免疫表型特征，经RT-PCR、FISH及全基因组测序检测均未发现PML-RARα融合基因及其他RAR基因重排，诊断为“APL样白血病”，对ATRA和ATO治疗无反应，采用标准IA方案诱导化疗1个疗程后达到血液学缓解。

本例患者检出t（16;17），在白血病中是一种罕见的染色体异常形式。检索国内外文献仅见Bhat等[10]于2016年报道首次发现16及17号染色体易位APL患者，该病例为13岁男性患者，因齿龈出血就诊，骨髓发现90％少颗粒型早幼粒细胞，染色体为t（16;17）（q12;p13），实时定量PCR及凝胶电泳的方法均未检测到PML-RARα融合基因，包括bcr1、bcr2和bcr3形式的杂交转录物。该患者接受了IC-APL方案联合ATRA诱导化疗并出现显著的治疗反应。本文报道病例为t（16;17）（q11;p12）染色体改变，与上述文献中所报道患者染色体易位位点接近，同样未出现PML-RARα融合基因，且使用二代测序高通量融合基因筛查方法及全基因组测序均未检测出APL相关的其他融合基因，所检测到的数个融合基因均非16及17号染色体易位所产生，分析其原因为在16号和17号染色体的断裂点可能处于非编码区，未产生基因的改变。

除APL外，16、17号染色体平衡易位在白血病及非血液系统疾病中均仅有个例报道，薛永权等[11]于1989年报道1例伴有t（16;17）（q12;q25）的骨髓增生异常综合征患者，后转变为红白血病并出现其他染色体异常；仇惠英等[12]于2009年报道1例伴有t（16;17）（q24;q12）的难治性AML-M_2a_患者，经标准联合化疗及单倍体造血干细胞移植均治疗无效，且移植后早期即出现髓外侵犯。陈艳等[13]曾报道1例包含t（16;17）（p13;q22）的复杂核型的混合表型白血病病例，巢式RT-PCT检测到SET-NUP214融合基因转录本。上述后2例患者及本文患者均在发病早期即发生髓外侵犯，且均有淋巴结肿大。髓外侵犯是AML的一种预后不良因素，易复发，且中枢神经系统侵犯发生率较高，通常在CR_1_期进行异基因造血干细胞移植，可获得生存获益[14]。但到目前为止，仍无数据说明t（16;17）与髓外侵犯的关系。此外，国内外文献报道伴有t（16;17）平衡易位的非血液系统疾病病例包括孕检时羊膜腔穿刺、羊水细胞培养发现胎儿染色体异常[15]，少精症男性外周血染色体检查发现[16]，习惯性流产[17]，遗传性神经病与压力性麻痹[18]，动脉瘤骨囊肿[19]，深部纤维组织细胞瘤[20]等。以上病例均与本文病例染色体易位位置不同。

综上所述，伴有t（16;17）的骨髓APL样形态学改变的白血病较为罕见，此前仅有1例报道，与本文病例均无融合基因检出。因病例数少，该类型白血病的预后情况尚不能明确，本病例存在包括淋巴结、骨、中枢神经系统的髓外侵犯，对AML的化疗方案敏感，异基因造血干细胞移植前评价骨髓及髓外病灶，已达完全缓解状态，造血干细胞移植后获得了7个月的持续缓解，但最终出现了髓外复发。
